# Training pet dogs for eye-tracking and awake fMRI

**DOI:** 10.3758/s13428-019-01281-7

**Published:** 2019-07-16

**Authors:** Sabrina Karl, Magdalena Boch, Zsófia Virányi, Claus Lamm, Ludwig Huber

**Affiliations:** 1 Clever Dog Lab, Comparative Cognition, Messerli Research Institute, University of Veterinary Medicine Vienna, Medical University of Vienna, University of Vienna, Vienna, Austria; 2 Social, Cognitive and Affective Neuroscience Unit, Department of Basic Psychological Research and Research Methods, Faculty of Psychology, University of Vienna, Vienna, Austria; 3 Department of Cognitive Biology, Faculty of Life Sciences, University of Vienna, Vienna, Austria

**Keywords:** Domestic dog, Eye-tracking, Functional magnetic resonance imaging, Positive reinforcement, Dog training

## Abstract

In recent years, two well-developed methods of studying mental processes in humans have been successively applied to dogs. First, eye-tracking has been used to study visual cognition without distraction in unrestrained dogs. Second, noninvasive functional magnetic resonance imaging (fMRI) has been used for assessing the brain functions of dogs in vivo. Both methods, however, require dogs to sit, stand, or lie motionless while yet remaining attentive for several minutes, during which time their brain activity and eye movements are measured. Whereas eye-tracking in dogs is performed in a quiet and, apart from the experimental stimuli, nonstimulating and highly controlled environment, MRI scanning can only be performed in a very noisy and spatially restraining MRI scanner, in which dogs need to feel relaxed and stay motionless in order to study their brain and cognition with high precision. Here we describe in detail a training regime that is perfectly suited to train dogs in the required skills, with a high success probability and while keeping to the highest ethical standards of animal welfare—that is, without using aversive training methods or any other compromises to the dog’s well-being for both methods. By reporting data from 41 dogs that successfully participated in eye-tracking training and 24 dogs IN fMRI training, we provide robust qualitative and quantitative evidence for the quality and efficiency of our training methods. By documenting and validating our training approach here, we aim to inspire others to use our methods to apply eye-tracking or fMRI for their investigations of canine behavior and cognition.

**Electronic supplementary material:**

The online version of this article (10.3758/s13428-019-01281-7) contains supplementary material, which is available to authorized users.

After primates (see, e.g., Tomasello & Call, 1997), rodents (Pineno, 2010), corvids and parrots (ten Cate & Healy, 2017), and cetaceans (Mann, 2018), canines have become the main model system for the investigation of cognitive behavior in nonhuman animals (see, e.g., Katz & Huber, 2018; Miklósi, 2014). From a scientific point of view, dogs have become particularly attractive because of the interesting but yet not well understood interplay of long-term (canine evolution) and short-term (domestication) phylogenetic as well as ontogenetic (lifetime experiences) influences on cognition and behavior. After several decades of assuming both a special sensitivity and also cognitive ability of understanding humans (see, e.g., Huber, 2016) due to domestication and an increased dependence on humans (e.g., Hare & Tomasello, 2005), a current trend in the attempts to explain dog cognition and behavior is to emphasize socio-ecological factors (changed feeding ecology and social organization) and remaining traits from their wild progenitor, the wolf (Marshall-Pescini, Cafazzo, Virányi, & Range, 2017; Range & Virányi, 2015). In addition, the enormous amount of experience during their life with humans, which is often characterized by close, intimate relationships, must not be underestimated (Udell & Wynne, 2008). From an applied point of view, dog research is producing a great impact on society, spanning a range from how to handle man’s best friend as pets, or even as therapists to “bad dogs” (dog biting), let alone the practical importance of a better understanding of dogs for the growing number of industries (e.g., scent detection) that utilize the behavior of domestic dogs.

So far, the methodology for the investigation of dog cognition has been based predominantly on behavioral experimentation or observational studies. The great majority of studies have relied on analysis of the performance of dogs when they are confronted with challenging tasks in either the physical or the social domain (Bensky, Gosling, & Sinn, 2013; Lea & Osthaus, 2018). Only a few studies have applied advanced psychophysical techniques to examining perceptual and cognitive abilities with the aid of highly controlled, experimentally manipulated stimulation. An example of such a sophisticated stimulus device is the touchscreen (Steurer, Aust, & Huber, 2012; Wallis et al., 2017), which has been used to test discrimination, categorization, concept formation, and even inferential reasoning (Aust, Range, Steurer, & Huber, 2008; Range, Aust, Steurer, & Huber, 2008; Wallis et al., 2016). However, the use of more naturalistic stimuli, such as humans showing specific behavior, facial expressions, or gestures (e.g., pointing live, presentation), requires measuring the dog’s looking behavior via its head or eye movements (e.g., Adachi, Siebrits, Peirce, & Desroches, 2007; Barnard et al., 2016; Faragó et al., 2010; Huber, Racca, Scaf, Virányi, & Range, 2013; Mongillo, Scandurra, Kramer, & Marinelli, 2017; Racca et al., 2010; Schmidjell, Range, Huber, & Virányi, 2012; Wallis et al., 2015). These orientation movements that signal looking preferences, attention patterns, or gazing have usually been examined with video analysis—that is, by recording the dog’s head movements with video camera(s) and subsequently “manually” coding the video files using behavioral event recording software such as The Observer XT (Noldus Information Technology, The Netherlands) or Solomon Coder (developed by András Péter, www.solomoncoder.com).

These traditional methods are, however, not fine-grained enough to reveal the subelements of the looking behavior that are necessary to uncover the underlying mental mechanisms of the performance (Quinn, Doran, Reiss, & Hoffman, 2009). When only head movements are used, it is impossible to define to which parts of human faces the dogs’ attention is drawn (Somppi, Törnqvist, Hänninen, Krause, & Vainio, 2012). However, the quality of the technology or methods strongly depends on the research question, so that the sophisticated, mostly much more expensive methods only make sense if the rough methods are unable to answer the question. For instance, a video camera placed in front of the dog’s head can measure whether the dog is looking left or right, which is sufficient if the stimuli are distantly positioned in the left and right viewing fields of the dog. Examples are studies about size constancy (Müller, Mayer, Dörrenberg, Huber, & Range, 2011), looking preference for novel objects, face discrimination and inversion responses (Racca et al., 2010), and looking preferences for emotional human stimuli (Racca, Guo, Meints, & Mills, 2012). In conclusion, the choice of the methods depends on the necessary accuracy for measuring of the dog’s looking behavior. Only with the measurement of the dog’s eye movement with high spatial and temporal resolution one can determine how the dog scans the human face, for example, including fixations and quick shifts.

In human psychology, eye movement tracking has been developed as a technique for directly, objectively, and accurately assessing human gazing behavior (for an overview, see Holmqvist et al., 2011). For example, researchers aimed at determining the patterns of human face scanning by measuring frequencies, durations, and probabilities of fixations. The resulting spatial and temporal characteristics of fixation sequences could be used to examine human face perception (Walker-Smith, Gale, & Findlay, 1977) or the cognitive development of joint attention (Carpenter & Tomasello, 1995).

Three decades later, eye-tracking has also found its way into research on nonhuman animals. It was first used in veterinary medicine (neuro-ophthalmology) to diagnose ocular motor abnormalities such as nystagmus. For this purpose, the heads of untrained dogs were stabilized and held rigidly by locking arms or velcro head harnesses (Dell’Osso, Williams, Jacobs, & Erchul, 1998; Jacobs, Dell’Osso, Wang, Acland, & Bennett, 2009). Primatologists had been the first to recognize the advantage of eye tracking in monkeys (Guo, Robertson, Mahmoodi, Tadmor, & Young, 2003) and apes (Kano & Tomonaga, 2009, 2010) that are neither rewarded (as in conditioning paradigms) nor restrained (as in some preferential-looking paradigms), and therefore could show more natural behavior. The first attempt to apply eye-tracking in dog research utilized a head-mounted, portable, video-based eye-tracking camera. Williams, Mills, and Guo (2011) modified a VisionTrak head-mounted eye tracker (ISCAN ETL 500; Polhemus, Vermont, USA) to be used with one dog that was trained to wear the apparatus. Nowadays, lightweight goggles with in-built infrared cameras, such as the Tobii Pro Glasses 2 (Tobii AB, Stockholm, Sweden), are available for humans, but so far they do not work with dogs.

Contact-free eye-tracking with dogs has utilized standard, table-mounted eye-tracker systems with a remotely placed infrared camera. Such systems measure the dogs’ eye movements using infrared corneal reflection techniques. In most studies, the camera was integrated below a computer monitor placed at some distance from the dogs’ eyes, such as an iView X RED (SensoMotoric Instruments GmbH, Germany; Somppi et al., 2012, Somppi, Törnqvist, Hänninen, Krause, & Vainio, 2014; Somppi et al., 2016; Törnqvist et al., 2015), Tobii X50 (Tobii AB, Stockholm, Sweden; Téglás, Gergely, Kupán, Miklósi, & Topál, 2012), or EyeLink 1000 (Barber, Randi, Müller, & Huber, 2016). In these first studies using eye-tracking for dogs, the researchers investigated how dogs perceive human gestures in different ostensive contexts (Téglás et al., 2012), how dogs look at actual objects within pictures that differ in terms of novelty and categorical information (human and dog faces, toys, alphabetic characters; Somppi et al., 2012), whether dogs show a human-like facial inversion effect (Somppi et al., 2014), and how dogs with different social experience (pet dogs vs. kenneled dogs) look at pictures showing interactions between humans and dogs (Törnqvist et al., 2015). In five studies, the focus was on the dog’s response to emotional expressions. Researchers asked whether dogs show an attentional bias toward threatening social stimuli and whether their gaze fixation patterns are influenced by the different facial areas of human and dog faces (Somppi et al., 2016), how dogs with different experience with humans (pet vs. laboratory dogs) scan human emotional faces (Barber, Müller, Randi, Müller, & Huber, 2017; Barber et al., 2016; ), whether oxytocin has an impact on the processing of human facial emotions (Kis, Hernádi, Miklósi, Kanizsár, & Topál, 2017; Somppi et al., 2017), and whether the latter effect correlates with cardiac responses (Barber et al., 2017). Most recently, researchers have investigated con- and heterospecific auditory–visual matching in dogs when seeing a woman’s face and hearing her voice or seeing a dog’s face and hearing its barking (Gergely, Petró, Oláh, & Topál, 2019).

A crucial feature of investigating response patterns to such sophisticated stimuli by means of eye-tracking is that (1) the animals need to be in a relaxed enough condition to pay attention to and process the stimuli presented, and (2) at the same time, they need to stay motionless so that their head does not move throughout calibration and validation, as well as during the whole subsequent sequence of stimulus presentations. Calibration is used to collect fixation samples from known target points in order to map the raw eye data to gaze positions. Targets like white disc patterns on the black screen or even animated images are presented serially on a screen. The dog fixates each while samples are collected, and feedback graphics are presented on the host PC display. The calibration is checked automatically when it is finished, and diagnostics are provided. The subsequent validation provides the experimenter with information about calibration accuracy. This is measured in terms of the difference between the computed fixation position and the fixation position for the target obtained during calibration. This error reflects the gaze accuracy of the calibration.

It is obvious that the gazing patterns of subjects that are stressed by being restrained or forced to perform the task will not provide reliable data on how animals process the pictures, videos, and other visual stimuli they are presented with (Niehorster, Cornelissen, Holmqvist, Hooge, & Hessels, 2018). Moreover, physical fixation, such as being harnessed (Jacobs et al., 2009) or kept still by a human (i.e., the experimenter or the caregiver restrains the dog’s body or head manually), likely compromises both the natural looking behavior of dogs and their welfare. Habituating animals to such treatments requires intensive training that, we argue, is better to invest in getting reliably motionless and attentive subjects without any use of physical restraint. Not only may the well-being of the dogs favor this solution but, as long as pet dogs are being tested, the availability of subjects also may increase with this approach, which dog caregivers are likely to prefer.

An even more radical step forward in assessing cognitive processes as well as their neural correlates noninvasively in awake dogs is the use of functional magnetic resonance imaging (fMRI). Although dogs have been tested in behavioral studies of how they solve challenging problems or interact with humans, when perceiving human gestures, expressions, or even voice, we are limited in our conclusions about the underlying cognitive processes. It is not enough to infer from behavior what dogs think, how they feel, and what they understand. And we do not know whether similar behaviors across species result from the same proximate mechanisms. Neuroimaging provides a first look into the working brain during perception and the subsequent mental processes.

The advantages of this neuroimaging technique of estimating brain activity by changes in hemodynamic responses are at least threefold: It can localize neural activity in the brain with high precision, it allows network-level analyses, and, if applied properly, causes no harm nor requires invasive procedures in the tested subject. However, this comes at a cost. For instance, MRI data are highly susceptible to corruption from subject motion. The precise spatial localization of neural activity in the relatively small dog brain therefore requires that the dog lie motionless in the noisy, vibrating, and spatially restrained MRI scanner bore. Because anesthesia or sedation would negatively affect both brain function and cognition, by impeding attentiveness, altering the state of consciousness, and reducing rates of blood flow and respiration (Thompkins, Deshpande, Waggoner, & Katz, 2016), alternative ways are needed to achieve stillness. For the same reasons we described for eye-tracking, testing animals that, due to their training, stay in the scanner on a voluntary basis is highly preferred over physical restriction. Yet not only from an experimental perspective, but also from an ethical one, dogs must not be restrained but be free to leave the scanner whenever they want.

A breakthrough in training animals to remain still, wakeful, and attentive during scanning was achieved only a decade ago (Berns, Brooks, & Spivak, 2012; Tóth, Gácsi, Miklósi, Bogner, & Repa, 2009), and soon it was envisioned as a proper, noninvasive research technique to understand the neural mechanisms of canine cognitive function.

So far, five independent research groups—two in the USA (Atlanta, GA, and Auburn, AL), one in Mexico (Querétaro), and two in Europe (Budapest, Hungary, and Vienna, Austria)—have captured brain images of nonsedated and largely unrestrained dogs, and their work and publications indicate the interest in and the importance of this new frontier in functional neuroimaging (see Andics & Miklósi, 2018; Berns & Cook, 2016; Bunford, Andics, Kis, Miklósi, & Gácsi, 2017; Cook, Brooks, Spivak, & Berns, 2016; Huber & Lamm, 2017; and Thompkins et al., 2016, for reviews). Starting with studies on reward processing (Berns et al., 2012; Berns, Brooks, & Spivak, 2013; Berns, Brooks, Spivak, & Levy, 2017; Cook, Spivak, & Berns, 2014), subsequent studies investigated the default mode network (Kyathanahally et al., 2015), olfactory processing (Berns, Brooks, & Spivak, 2015; Jia et al., 2014; Jia et al., 2015), face processing (Cuaya, Hernández-Pérez, & Concha, 2016; Dilks et al., 2015; Thompkins et al., 2018), response inhibition (Cook, Spivak, & Berns, 2016), auditory processing (human and dog vocalizations: Andics, Gácsi, Faragó, Kis, & Miklósi, 2014; human words: Andics et al., 2016; Prichard, Cook, Spivak, Chhibber, & Berns, 2018), and emotion processing (“jealousy”; Cook, Prichard, Spivak, & Berns, 2018; human emotional faces: Hernández-Pérez, Concha, & Cuaya, 2018). These studies have not only provided a “proof of concept,” but also demonstrated the great potential of this neuroimaging approach to canine cognition. Still, a number of technological and methodological challenges need to be overcome to fully tap this potential (Huber & Lamm, 2017). Among them are appropriate training programs, which are both efficient and ethically responsible—that is, promoting rapid acclimation to the scanner environment with minimal stress and discomfort to the dogs. The challenge is to train animals to remain attentive and cognitively responsive without moving for long enough to make the necessary recordings. In eye-tracking, this means keeping the head still for at least 1 min, a time period that is needed to conduct the calibration and validation procedure and to record the dogs’ gazing patterns in response to the test stimuli presented. In the case of fMRI, a rule of thumb is that dogs need to stay still for at least 4 min, since this usually corresponds to the time period required to collect a sufficient number of fMRI images (with the actual duration depending on the imaging sequence and the experimental design).

So far, researchers who have successfully published studies about fMRI in awake dogs have used slightly different training methods that included chaining (e.g., Berns et al., 2013), target stick (e.g., Jia et al., 2014), and model–rival (e.g., Andics et al., 2014) training. Despite some differences that we will discuss later, all of them include techniques based on the principles of classical and operant conditioning (Dickinson, 1980). These denote learning processes in which new behaviors are acquired and modified through their association with consequences. All of these training methods, by strictly avoiding aversive methods, rely on reinforcing desired behaviors in order to increase the likelihood that the behaviors will occur again, as well as using negative punishment to decrease the probability of undesired behaviors. The training methods used so far have not been systematically compared; thus, we are far from describing a gold standard. Due to various unpredictable circumstances or to events unrelated to dog training—such as dogs that become sick, caregivers stopping participation for personal reasons, and so forth—we cannot compare the different variations quantitatively in terms of training success (e.g., the ratio of dogs that have been tested successfully of all dogs with which the researchers started training). Still, the overarching goal of any training methodology is to reduce training time while maintaining success in the desired behavior (Thompkins et al., 2016).

Here we aim to provide a comprehensive training program that has proved highly successful and, thus, can serve as a reference approach for future research. In short, this program is based on (a) systematic desensitization and habituation to the potentially stressful environment and (b) the shaping and ultimate chaining of several requisite behaviors by using primary and secondary reinforcers. In the case of fMRI, the dogs need to be habituated to the very loud MRI scanner noise (sound pressure levels of up to 96 dB), the operating vibrations caused by the magnet, the tight scanner enclosure (a constricted tube that may provoke apprehension in animals with enclosure anxiety), and the scanner ramp to get onto the elevated and narrow “patient table.” In case of eye-tracking, habituation is less of an issue, but shaping the necessary behaviors, such as putting the head on the chin rest and sitting or standing still, represents a similar training challenge.

## Method

### Ethics

All experimental procedures described here were discussed and approved by the institutional ethics and animal welfare committee in accordance with Good Scientific Practice (GPS) guidelines and national legislation at the University of Veterinary Medicine Vienna (approval number: 09/08/97/2012). In the case of the fMRI training, the decision was made on the basis of a pilot study at the University of Vienna.

### Dog training for accurate eye-tracking

#### Subjects

##### Recruiting

All subjects were privately owned pet dogs recruited from human caregivers in Vienna via our Clever Dog Lab (CDL) website and database. The pet dogs were of various breeds, of both sexes (16 males, 25 females), and their ages ranged from eight months to nine years when they started the training (see Table S1). Most dogs were participating in dog activities such as agility, dog dance, therapy dog training, man trailing, search and rescue dog training, dummy training, training in obedience classes, and so forth, at least one or two times a week and were experiencing individual dog training on a daily basis by their caregivers. Their caregivers gave written consent for them to participate in the study.

##### Suitability check

Before starting the training, we checked whether the dog was suitable for the eye-tracker task. Limiting factors for choosing the subjects were, for example, age (maximum 10 years old) and eyesight, the eye shape, general state of health, length of hair around the eyes, excitement level of the dog, and whether the eye tracker could track the dog’s eyes. We needed to be sure that the dogs were able to see the visual stimuli presented on the screen. Therefore, we made a rapid eye check with a flashlight to exclude cataracts. The shape of the dogs’ eyes was also important. If the eyes were too droopy or, as in some dog breeds, tended to have visible third eyelids, it could happen that the eye tracker would have problems detecting the pupil because of the additional reflection (of wet areas). Since the dogs should sit or stand calmly while doing the eye-tracking, they should be in good health condition so they could repeat the procedure for several minutes. If the hair or the eye lashes around the dog’s eye were too long, this might also have distracted the eye-tracker system from detecting the pupil. The color of the iris could influence the pupil detection, as well. If the color of the dog’s eye was very bright—for instance, light blue—or the edge of the pupil was not really clear, the eye-tracking system could hardly distinguish between the iris and the pupil itself. The last suitability criterion was that the dog should be able to behave calmly and conduct a task during which it needed to be almost motionless for a certain amount of time (maximum of up to 3 min per trial). If all crucial criteria were fulfilled by the dogs, the training started and took place in the Clever Dog Lab, Vienna, at least once a week.

##### Study sample

The sample of subjects used for training and finally for the eye-tracking studies in Vienna consisted of 41 pet dogs (Table S1). All of them have been trained with a big screen, and out of these ones, 30 dogs learned to perform eye-tracking tests on a small monitor, as well (see below and Table S2).

#### Experimental setup

The dog training took place in the eye-tracking room of the Clever Dog Lab, Messerli Research Institute, at the University of Veterinary Medicine Vienna. The eye-tracking room is a large (588 × 356 cm), quiet, windowless room equipped with the chin rest device and the eye tracker (see Fig. S1). Light conditions in the room were kept constantly at 75 lux using LED light bulbs (9.5 W, 2700 K Philips GmbH Market DACH, Germany). We used the EyeLink 1000 eye-tracking system (SR Research, Ontario, Canada) because it allows a maximum amount of flexibility with regard to both data analysis and stimulus presentation. Because the camera was sitting just below the tracked area the subject was viewing, it could be used with life-sized stimuli being back-projected onto a large projection screen, or a computer screen, or even with live presentation. For details about the system, see Barber et al. (2016). Of course, other systems, such as the Tobii system or the iView X RED, can be used as well.

The maximum head movement the EyeLink 1000 could track without accuracy reduction was 25 mm horizontal and vertical, and 10 mm back and forth. The setting included a chin rest for stabilizing the participant’s head, either a big or small screen encompassing stimuli display area in the middle, and an eye movement recording camera connected with an infrared illuminator to its right.

**Fig. 1:**
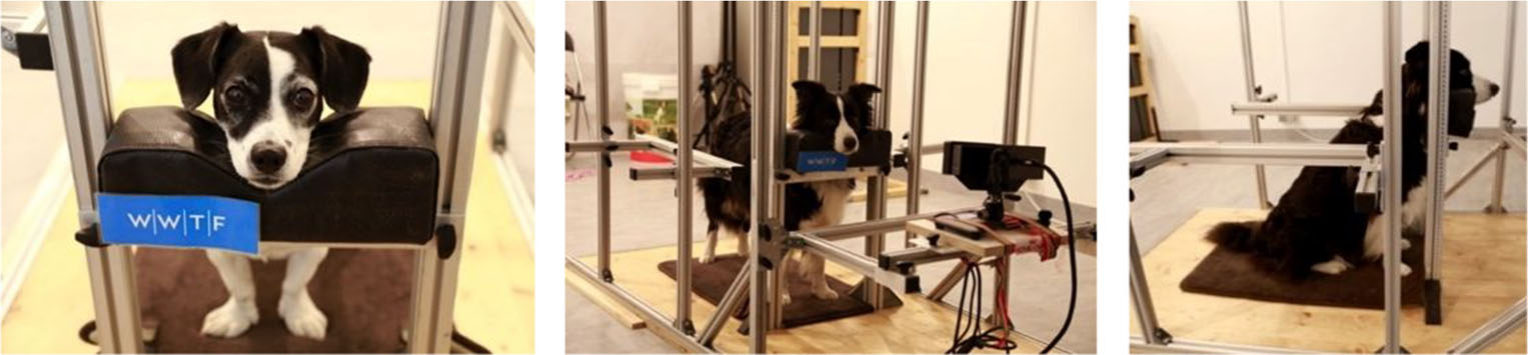
Training snapshots demonstrating the correct position of the head on the chin rest of a standing (middle) or sitting (left, right) dog

We used a customized chin rest device for head stabilization (Fig. 1). A pillow with a v-shaped depression was mounted on a frame, to allow vertical adjustment of the chin rest to the height of the individual dog. The frame consisted of aluminum profiles (©MayTec, Dachau, Germany) that allowed the easily adjustable but stable fixation of additional equipment (e.g., cameras). The chin rest was positioned at a distance of 200 cm from a big projection screen (200 × 200 cm) and 50 cm from a small computer monitor (display PC monitor, 27-in., Asus PB 278), the camera and infrared illuminator. The precise height of the camera and chin rest and the angle of the camera were adjusted to each participant.

**Fig. 2:**
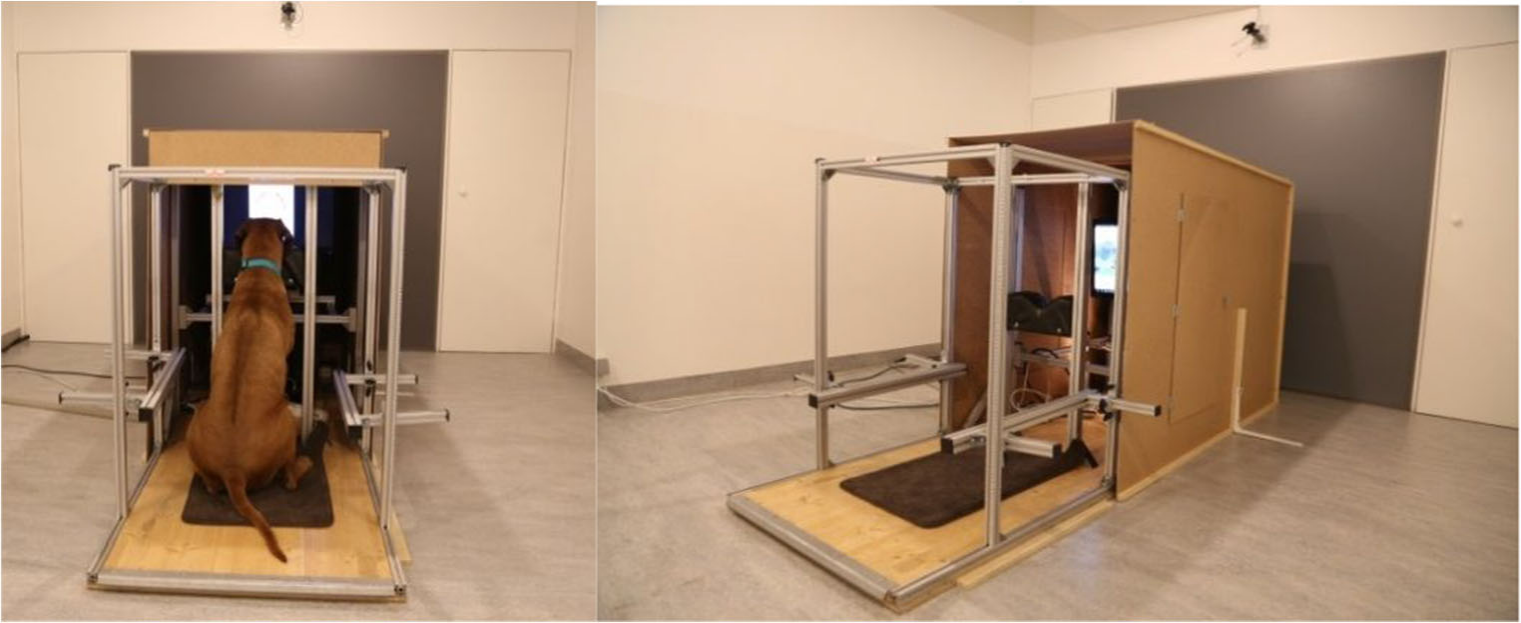
A dog watching a video on the small monitor (left), and a side view of the “dog cinema” (right)

We built a wooden box (170 × 120 × 84.5 cm) around the eye-tracker stand as a means to reduce the dogs’ distraction (Fig. 2). This box (which we refer to as the “dog cinema”) had several doors (44 × 44 cm) in the side walls (75 × 40 cm), to be able to check the dog’s behavior inside, to give treats, and to adjust the eye tracker before each training or test session.

#### Procedure

The participating dogs were trained at least once a week and each training session lasted approximately 30–45 min—including breaks, depending on the dog’s condition.

During the entire dog training we used a clicker (small device that produces a metallic click sound) as a secondary reinforcer and worked with positive reinforcement of the correct behavior. The clicker marked the correct behavior of the dog and the reward for the dog followed immediately. This enabled us to announce the following reward for the dog even over a distance or when we were outside of the eye-tracker room. The reward used was dry food, and pieces of sausage (or another higher-quality food like cheese, depending on subject preferences or allergies) were used as “jackpot treats”—for instance, for outstanding performance or fast improvement, or to increase the dog’s motivation.

First, all potential eye-tracking dogs were trained to be able to perform the calibration and validation procedure and to perform eye-tracker tests with a big screen, following a slightly different training protocol. The dog training process for using the eye tracker and a small computer monitor in front of them consisted of three basic phases: (1) chin rest training, (2) white disc pattern presentation on the monitor training, and (3) calibration and validation training with the eye-tracker system.

#### Phase 1: Chin rest training

The first phase of training began with teaching the dog to remain calm at the chin rest. This process started with so-called free shaping to form the correct behaviors in the dogs. The dog to be trained was free to move around the eye-tracker apparatus and familiarize himself with the room and the equipment first. If necessary, the dog could be lured—for instance, with food or pointing gestures—into the apparatus at the beginning. At this stage it was important to observe the dog’s reactions. If it showed any kind of avoidance or fear-related signals, training could be modified in order to comfort the dog and reward him for approaching and interacting with the apparatus. Dogs were then guided—for example, with hand signals—toward the chin rest, and reinforced once any interest in the chin rest was expressed. If needed, dogs were initially lured over the chin rest, but then they often offered to lay their head on it on their own. Free shaping was then used to gradually increase the time while resting. To train the dog to reliably lay its head on the chin rest, we established a hand signal first. When the dog entered the apparatus after the cue, the clicker marked the correct behavior and the treat was given immediately thereafter. In addition to the hand signal, when the behavioral response was provided reliably, a vocal signal was introduced and was used to verbally send the dog to the chin rest (“Rest”). To train the dog to remain in the apparatus and stay still for longer periods, the dog was reinforced by gradually increasing the rest time. To generalize this behavior, we began to move around the dog and slowly increased the distance until we were able to completely leave the room. At the end of the chin rest training phase, the dog was required to stay calm on the chin rest for up to 1 min without moving even when distracted—as, for instance, by outside noise.

To make the situation most comfortable for the dog, the subjects were allowed to either sit or stand at the chin rest (Fig. 1). Age, health, physical condition, and overall well-being were considered when making this determination. For example, older dogs would likely prefer to sit. Therefore, during training this behavior was reinforced right from the beginning, to help them be more comfortable. When the dog chose the sitting position, it was necessary to regularly control for the dog’s head and body location. For example, while sitting it could happen that the nose was tilted upward, especially when the dog sat too far away from the chin rest, which could result in inaccurate detections or results in the eye-tracking. In comparison, when the dog stood at the chin rest, the head was more straight and to the front. Adaptations such as lowering the chin rest or training the dog to sit closer to the chin rest were especially helpful. Note that the prone position of the dog in the eye-tracker apparatus is not recommended, since it would likely encourage the dog to fall asleep.

#### Phase 2: White disc pattern presentation training on the display PC monitor

To accustom the dogs to the new equipment, the display PC monitor was first placed 100 cm away from the dog’s head. We used a variety of animal videos to facilitate the dogs’ attention to the monitor (Fig. 2). To prepare the dogs for the calibration and validation procedure, we created a special screen presentation with Microsoft PowerPoint 2010. The presentation consisted of several black slides with a big (diameter: 7.5 cm) white-disc pattern, placed at different positions on the screen. The dogs learned a certain vocal signal (“Guck”) to look at the monitor and were immediately rewarded when they seemed to gaze at the white disc pattern. The trainer was outside the eye-tracking room and presented the stimulus on the monitor while she controlled for the dog’s eye movements on the camera setup screen next to her. As soon as the dogs were used to the monitor at that distance, we placed it right behind the eye-tracker camera (distance, chin rest to monitor: 50 cm). Then we decreased the size of the white disc pattern (from 7.5 cm to 2 cm in diameter) in a step-wise fashion and presented it in dynamic mode along a triangle-shaped trajectory (similar to the EyeLink three-point calibration mode). From our experience, a moving stimulus increased the dogs’ attention and motivation to gaze-follow it, as compared to a static one. Next, we presented the stimulus according to the EyeLink calibration/validation mode: namely a white disc pattern (diameter: 2 cm) with a black hole (diameter: 1 cm). This stimulus only appeared on the three positions of the triangle without any movement. During this training step, the dogs had to learn to stay focused and to gaze at the position of the white disc stimulus for at least 3 to 4 s at five to six different locations in a row. We estimated the accuracy of the gazing behavior with the camera setup screen of the eye-tracker system and motivated and rewarded the dog for accurate gazing with verbal praise during the training trial. At the end of each training trial, the dogs were rewarded with food. The next black slide, with the stimulus at a different position, was only shown after correct gazing behavior. At the end of Phase 2, we showed four to eight consecutively appearing white disc patterns and combined them with videos, to get the dogs used to the future experimental trials. We slightly increased the duration of the presented videos over sessions, and the dogs were rewarded at the end of each training trial.

#### Phase 3: Calibration and validation training with the eye-tracker system

In Phase 3, we started to practice the real calibration and validation procedure with the eye-tracker system and the display PC monitor. We repeated this training procedure until the system confirmed a successful calibration and the deviation from the validation points was minimal (less than 0.5 deg). To optimize the training progress and practicing of the calibration procedure, it would be possible to use animated targets—for instance, flickering or looming dots, designs, or pictures—instead of static dots. This might help to get the dogs’ attention faster and keep it longer. Therefore, this could eventually shorten the entire calibration/validation training process and the “refresh time” after a break between different studies. With the eye-tracking system, we took snapshots of the dogs gazing at the calibration points and checked whether these represented the shown key points of an isosceles triangle from the calibration mode. Then we again added animal videos in order to imitate a real test trial. We slowly increased the time of the videos shown to 45 s, to get the dogs used to staying longer in the chin rest. The dogs were rewarded after we had already stopped the videos, to avoid training effects on their watching patterns for future eye-tracker tests. It turned out that it was necessary to randomize the different training episodes (calibration, validation, calibration followed by validation and watching videos) to prevent the dogs from estimating the duration of the training trials in order to stop looking or to change their position. If we repeated the same training episode too often, some dogs started to assess the length of the trial and stopped looking in order to get the treat earlier.

Finally, we introduced the wooden box (“dog cinema”) around the eye-tracker apparatus to reduce the dogs’ distraction. We slowly habituated the dogs to it by adding and then closing all parts of the box one by one, to avoid any fear reactions in the dogs caused by the sudden darkness (Fig. 2). Afterward, we practiced the whole procedure of calibration, validation, and watching videos on the monitor in the closed box. The rear side of the box, behind the dog, always remained open.

#### Statistics

To investigate whether there was an effect of age or sex on the number of training sessions in the “big-screen” (*N* = 41) and the “small-monitor” (*N* = 30) dog samples, we used generalized linear mixed models (GLMM; Baayen, 2008). We included the breed of the dogs as a random effect. The predictor variables with fixed effects were age (covariate) and sex (factor), and as a response variable we included the number of training sessions. We included no random slopes in the model. To test for the influences of age and sex, we initially compared the fit of the full model (i.e., a model with age and sex included) with that of a respective null model (i.e., a model including only the intercept, with age and sex excluded), on the basis of a likelihood ratio test. All models were fitted in R (version 3.4.4; R Core Team, 2015) using the function glmer provided in the R package lme4 (version 1.1-13; Bates, Mächler, Bolker, & Walker, 2015). Overdispersion was no issue (dispersion parameters: big screen, 1.05; small screen, 0.53). We determined confidence intervals using the function bootMer in the lme4 package, and model stability by dropping the levels of the random effects one at a time and comparing the estimates obtained with those obtained for the full data set, which revealed no influential levels of the random effect.

### Training for fMRI in awake dogs

#### Subjects

In autumn 2017, after a pilot phase with five dogs, we began to train a cohort of 24 dogs (see Table S3). We used different breeds of dogs, and their ages differed from three months to nine years when they started the training (nine males, 15 females; see the supplementary material). Nineteen of these 24 dogs had already been successfully trained for eye-tracking studies before. The dogs needed to be healthy and able to lie still while perceiving visual stimuli on a monitor in front of them. We restricted our sample to dogs of around middle size (shoulder height: 45–60 cm) in order to have roughly equally sized heads (head circumference 35–45 cm) that fit into a human knee coil (diameter: 17 cm) that we used to scan the dogs’ heads. Dogs with metal implants—for instance, after surgeries—had been excluded, except if these implants were nonmagnetic. Before starting the training for fMRI testing, we asked the caregivers to visit the Small Animals Clinic of the University of Veterinary Medicine Vienna to conduct a clinical check with their dog. This consisted of a general orthopedic and neurologic examination to assess the dogs’ health and reactions to sensory stimuli and to confirm their analgesia for future fMRI studies.

##### Study sample

Because we do not report the pilot phase with five dogs here, the sample of subjects used for the training to conduct fMRI studies in Vienna consisted of 24 dogs (Tables S3 and S4).

#### Experimental setup

The main part of the dog training took place in a specially equipped room (size: 683 × 368 cm) at the Clever Dog Lab of the Messerli Research Institute at the University of Veterinary Medicine Vienna. Only the final part of the training was conducted in the actual MRI scanner and scanner room of the Neuroimaging Center of the University of Vienna, located in the radiology ward of the Dental University Clinic of Vienna (see Fig. S3).

**Fig. 3:**
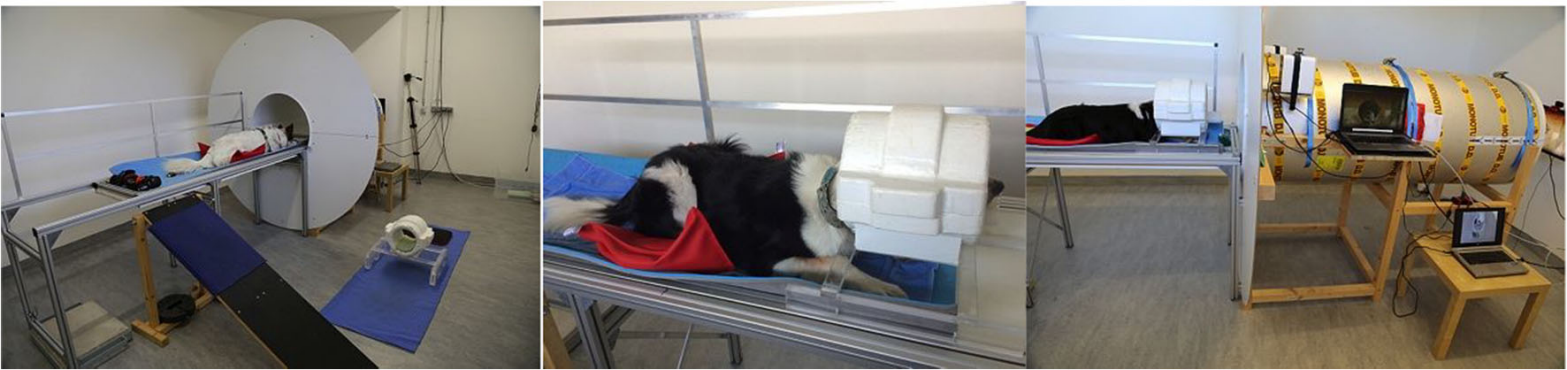
Mock scanner: (Left) Dog lying on the movable table in front of the mock scanner. (Middle) Dog in prone position with head in the mock coil. (Right) Patient table, mock bore, loudspeaker, laptops, and display monitor in front of the bore

The training room at the Clever Dog Lab was equipped with a so-called mock scanner, a replica of a real scanner that provides a realistic approximation of an actual MRI scanner, to allow acclimation and training of dogs in a controlled environment for a fraction of the cost of MRI access (Figs. 3, S2). Our mock scanner was built by our technical staff in our workshop at the Messerli Research Institute, designed to provide dogs with an experience as similar as possible to what they would experience in the real scanner. It consists of a moving table with a ramp; a realistic coil built of polyurethane (PU) hard-foam plates, replicating the human birdcage knee coil used for actual scanning; a 70-cm diameter tube simulating the bore, with cylindric entry and front facade panel (195-cm diameter); amplified speakers with subwoofer (Motiv B, Teufel, Germany), for realistic scanner noise production and vibration; and a 23-in. TFT screen (Samsung Syncmaster 2343NW) for visual stimulus presentation (Fig. 3). On top of the screen, a webcam (Logitech C525 HD) was installed that allowed us to see the dogs without approaching them from the front. All parts had been built with the identical measurements and were painted with colors similar to those of the respective parts of the real scanner, to provide an authentic scanning environment that would permit the dogs to gradually become accustomed to the future scanning procedure.

**Fig. 4:**
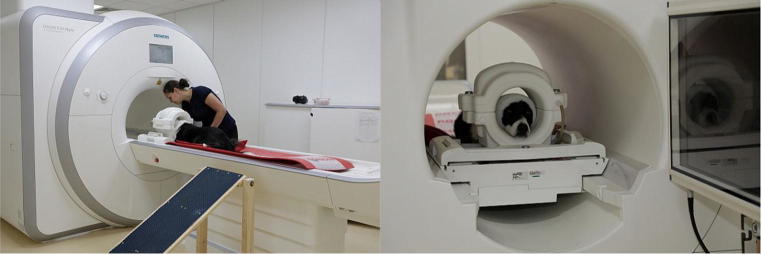
(Left) Dog lying in prone position on the patient table with its head in the human knee coil; the blue ramp was used for dogs to climb onto the patient table. (Right) The dog lying motionless with bandaged head in the knee coil in the MRI scanner bore

The scanner room at the Neuroimaging Center is equipped with a 3-T scanner (Siemens MAGNETOM Skyra) and an MR-compatible 32-in. screen (BOLDscreen 32 LCD for fMRI; Cambridge Research Systems; Fig. 4). First, to train the dogs and later on to scan their brains, we used a human Tx/Rx 15-channel knee coil (Siemens). The movable patient table was prepared with a slip-resistant mat and towels, to make the dogs more comfortable and to avoid having them slip or damage the table with their claws. Two sand-filled pillows on the sides were provided, to make the dogs feel more comfortable and stable in the prone position. The ramp to walk up to the scanner safely had a slip-resistant surface.

#### Training procedure

The training program for the dogs consisted of 17 pretraining steps with the mock scanner, and 12 steps with the real MRI scanner (Table 1). Importantly, the human caregivers remained near the dogs in the training environment during the entire training process. They either stayed in the adjoining scanner control room and could watch the training/testing or waited in the hallway, but they were always outside the actual scanner room.

**Table 1:** Learning outcomes (training criteria) of the 17 pretraining steps with the mock scanner and the 12 steps with the real MRI scanner

Training step	Training criterion
Mock scanner	
1	Learn to lie still in prone position on the floor
2	Learn to lie with the head in the mock coil and put paws under the coil holder
3	Learn to wear a flexible tube of fabric around the head and follow a vocal signal to put the head into the coil
4	Stay motionless with head in the coil on the floor for up to 5 min
5	Walk up and down the ramp
6	Lay down on the patient table
7	Stay still in prone position on the moving patient table
8	Stay still in prone position while being completely moved into mock scanner bore
9	Learn step by step to get used to wear human ear plugs (over sessions)
10	Habituate to gradually increased volume of different playback sounds of the future MRI scanner—up to 90–100 dB (over sessions)
11	Stay motionless with head in the coil in front of the mock scanner bore for up to 5 min
12	Stay motionless with head in the coil while being moved with the patient table
13	Stay motionless with head in the coil while being completely moved into mock scanner bore
14	Stay motionless with head in the coil in the mock scanner bore for up to 5 min
15	Stay motionless with head in the coil in the mock scanner bore while watching videos for up to 8–10 min (trainer periodically in dog’s field of view)
16	Stay motionless with head in the coil in the mock scanner bore while watching videos for up to 8–10 min (trainer stands entire period behind the dog)
17	Learn to wear an elastic bandage around the head
Real MRI scanner	
1a	Learn to wear a veterinary head bandage
2a	Habituate to the real scanner environment
3a	Transfer the mock scanner knowledge to the MRI scanner equipment
4a	Experience the scanner surrounding during a running scan
5a	Stay motionless with head in the knee coil in the MRI scanner bore for up to 5 min
6a	Stay motionless with head in the knee coil in the MRI scanner bore with scanner-loud playback sounds for up to 5 min
7a	Being fed in the scanner bore during a running scan
8a	Stay motionless with head in the knee coil in the MRI scanner bore while a real scan sequence starts
9a	Stay motionless with head in the knee coil in the MRI scanner bore while a set of real scan sequences consecutively start and run (functional scan)
10a	Stay motionless with head in the knee coil in the MRI scanner bore during a structural scan for 4–5 min (trainer in front of the dog/scanner)
11a	Stay motionless with head in the knee coil in the MRI scanner bore during a functional scan with monitor in front of the scanner
12a	Stay motionless with head in the knee coil in the MRI scanner bore during a functional scan while watching videos for up to 7 min

##### Pretraining with the mock scanner

At first, the dogs learned to stay calm in a prone position on a mat in front of the mock coil on the floor (Training Step 1 in Table 1). Then they were trained by using free shaping to put their head into the mock coil and their paws under the coil holder (Training Step 2 in Table 1). The dogs learned to wear a flexible tube of fabric around their heads in preparation for the prospective head bandage, and learned a specific verbal signal (“Rest”) to put their heads into the coil (Training Step 3 in Table 1). If a dog already knew a vocal signal for laying its head on something else beforehand, we used this individual vocal signal for it. While dogs were required to stay in this position, the resting time was slowly increased to up to 5 min (Training Step 4 in Table 1). The dogs learned to leave their heads motionless and at the same location. To help keep this head position in the coil, we carved different-sized PU hard-foam chin rests with a furrow for the dog’s snout (Fig. S7, right).

During the first training sessions, the dogs had to learn to walk up the ramp and were habituated to the manually moved patient table of the mock scanner (Training Steps 5–8 in Table 1). First, we slightly moved them back and forth, and then we moved the whole table into the mock scanner bore (without the coil). We established a verbal cue (“Achtung”) every time before we moved the table, to announce the following movement to the dogs and prepare them for it—especially when we moved the table backward out of the scanner from behind the dog.

Additionally, they learned step by step over several sessions to wear human ear plugs (EP5C, corded foam ear plugs; Blue Eagle, Taiwan; Training Step 9 in Table 1). From the beginning of the training, the dogs were acclimated to the slightly increased volume of different scanner playback sounds (Training Step 10 in Table 1). According to the dogs’ sense of well-being and behavioral reactions, we increased the volume in steps of five levels of sound intensity (volume 0–90 on the laptop; HP, Elite Book 840). If dogs showed any noise sensitivity, we increased the volume in smaller steps. We recorded the real scanner sounds beforehand, cut out the white noise, and started to play them scarcely audible. Over sessions, we increased the volume of the playback sound to up to 100 dB. In sessions in which we played back sounds of more than 50 dB, we applied ear protection to all dogs and humans present.

If the caregivers were experienced in dog training and willing to train their dogs the first steps themselves at home, as had been practiced at Emory University (e.g., Berns et al., 2012, 2013, 2015), we offered them the use of two different kinds of chin rests: initially a half-open one, and later a closed one like the mock coil (Figs. 3, S7). With those replicas, they could practice the mock coil procedure on the floor. Additionally, they received the scanner playback sound files, a pair of ear plugs, and a flexible tube of fabric to habituate the dogs to the noise and the equipment at home.

When the dogs were able to stay motionless in the mock coil on the floor for 5 min, they proceeded to work with the coil attached to the patient table. Thus, the next step for them was to stay calm in a prone position with their head in the mock coil in front of the mock scanner bore for up to 5 min (Training Step 11 in Table 1). At the end of this stage, the trainer started to slightly move around; that is, she left the dog’s field of view or operated the laptop behind the panel. Then the dogs were trained to remain still while they were moved with their heads in the coil into the mock scanner bore (Training Step 12 in Table 1). Initially, they were moved slightly back and forth. Again we used the vocal cue to announce the table movement to the dogs and then increasingly moved them completely into the mock scanner bore (Training Step 13, Table 1). They were trained to motionlessly stay in this position for up to 5 min (Training Step 14, Table 1). During this training stage, we played the different scanner playback sounds in a continuous loop and slowly increased the volume over time. As soon as they were patient enough to stay calm for 5 min in the mock scanner bore, we started to show videos on the screen mounted on the wall in front of the mock scanner. We began to simulate structural and functional scans by using the same playback sounds separately and practicing their usual durations (4–8 min). We established another vocal signal (“Aufpassen”) to announce the start of the scanner playback sounds, to prepare the dogs in the mock scanner bore. The dogs learned to lie still in the prone position with head in the coil in the mock scanner while watching videos for up to 10 min (Training Steps 15 and 16, Table 1). During this training section, the trainer varied between being in and outside the dog’s field of view in front of the mock scanner. The webcam on top of the monitor enabled us to observe the dogs while standing behind or next to the mock scanner bore without being seen by the dogs. Hence, at the end of the mock scanner training, we could stand behind the dogs as we would later, in the real scanner tests. During the last two training sessions, the dogs learned to wear an elastic bandage around their head, to get prepared for the veterinary head bandage they would have to wear in the real scanner (Training Step 17, Table 1). When the pretraining with the mock scanner was completed, the dogs advanced to the training in the real MRI scanner environment.

Seven caregivers had two participating dogs. Both of these dogs were brought to the training. While the first was trained, the second one was waiting next to the caregiver. Then the dogs changed roles/positions.

##### Training in the real MRI scanner

Before the dogs proceeded to the real MRI scanner training, the caregivers had been instructed about the MRI method application and had to fill out an MRI safety sheet about MRI-related details about their dogs. They received clear instructions about how to behave in the scanner environment with their dogs. Before entering the MRI scanner room, all metal-containing dog equipment was removed from the dogs, and they learned to wear a veterinary head bandage in addition to the ear plugs (Training Step 1a in Table 1). The head bandage assured that the ear plugs stayed in position, and the upholstery material enhanced the noise protection.

During the first training session in the scanner environment, we allowed the dogs to visually and olfactorily explore the MRI scanner room and equipment (Training Step 2a in Table 1). Then we started to repeat the training steps that had been exercised with the mock scanner (Table 1). Most of the dogs were immediately able to stay calm with their head in the knee coil in the MRI scanner for 3–5 min while they heard playback sounds within the first one or two sessions (Training Step 3a in Table 1). To train the dogs with the playback sounds before starting the actual scans, we used the patient intercommunication system of the scanner. The sounds were played with the same amplitude as the real scanner noise. The dogs had no problem at all remaining still on the electrically moveable patient table (Fig. 4). An important final step of the first training session with the real scanner was the acclimation to the magnet. Here the dogs had been led around the scanner and then rewarded with food in the scanner bore next to the coil while an actual scan sequence was conducted (Training Step 4a in Table 1). This was done to provide them with experience of how it feels to be near and in the magnetic field of the scanner.

Typically, after the first two to three training sessions (including Training Steps 5a & 6a in Table 1), the dogs were ready to experience their first real functional scan sequences while lying with their heads in the knee coil. During the first actual scans, the dogs were fed with pieces of sausage in order to create a positive association with the scan situation (Training Step 7a in Table 1). Some dogs reacted very sensitively to the scan itself or to the start of the scan sequence(s), and were tempted to leave the scanner. In these cases, we masked the real scan with the playback sound of the same scan and started the real scan delayed. Thus, the dogs felt more comfortable hearing the playback sounds and then had an easier time overcoming the simultaneous onset of the real scan. For advanced training phases and the actual data collection, we continued close monitoring of potential reactions to the onset of each sequence, to make sure the dogs showed no startle responses. The feeding during the scans was reduced in a step-wise manner until the dogs only got a reward at the end of a scan trial (Training Steps 8a & 9a in Table 1).

For the next training criterion, the dog was required to stay completely motionless throughout the whole structural scan (4 min). This was necessary in order to get an accurate structural scan of the dog’s brain and head. During the structural scan, the trainer was in front of the dog and could control for any movement of the dog (Training Step 10a in Table 1). After we had achieved that, we continued to train the dogs to remain still during a functional scan and to look at the monitor in front of them (Training Step 11a in Table 1). In this training phase, the dog also learned to accept the trainer disappearing from the dog’s field of view. At the end, the trainer stood behind the dog next to the patient table for the entire scan. The final goal before conducting fMRI tests was for the dogs to be able to remain motionless during a functional scan while watching videos for up to 7 min (Training Step 12a in Table 1, Fig. 4). During the last functional scans with video presentations before the actual tests and the fMRI tasks, we used an EyeLink 1000 Plus (SR Research) eye-tracking system to control live for eye movements (dogs’ attention) and/or in case the dogs tended to fall asleep. Additionally, we recorded the dogs with an action camera (Rollei, Bullet HD 4S) standing ~ 1 m in front of them throughout the whole training and testing, to double check their behavior.

During the fMRI training sessions, only the trainer was present in the scanner room. She stood silently and motionless behind the scanner bore and did not interact with the dog. The caregivers were either watching the training through the window of the scanner control room or waiting in the clinic hallway with the second dog.

#### Statistics

To test whether there was an influence of the age or the sex of the dogs on the number of training sessions (approximately 45 min each, including breaks) they needed to reach the training goal, we fitted a GLM with a negative binomial error structure and log link function (McCullagh & Nelder, 1989; a corresponding Poisson model was slightly overdispersed, with a dispersion parameter of 1.475)*.* To assess the significance of the full model (Forstmeier & Schielzeth, 2011), we ran a likelihood ratio test (Dobson, 2002), comparing it with the null model including only the intercept. We included the number of training sessions as the response variable, and age (as covariate) and sex (as factor) were included as predictor variables with fixed effects. Overdispersion was not an issue for this model (the dispersion parameter was 1.195). We assessed collinearity (Field, 2005) by using the vif function in the R package car (Fox & Weisberg, 2011) and found no issue (maximum variance inflation factor = 1.04). We assessed model stability by means of leverage values and the DFBbeta values (Field, 2005; G. P. Quinn & Keough, 2002), which revealed no obviously influential cases. We determined confidence intervals using the R function confint.glm. The sample size used in the model was 20 dogs. During the second analysis, we fitted a GLM with the same error structure and variables as above to only a subset, comprising the 16 Border Collies of the sample (see Fig. 6 below and Table S7). All models were fitted in R (version 3.4.4; R Core Team, 2015) using the function glm.nb in the package MASS (Venables & Ripley, 2002).

## Results

### Eye-tracking

Out of the 66 dogs we had initially invited, eight dogs were excluded due to their bad eyesight and/or health condition (*N* = 5), too much excitement (*N* = 1), too little motivation (*N* = 1), or fear of the apparatus (*N* = 1). We excluded 12 additional dogs during the training process because of either too much panting (*N* = 3) or barking (*N* = 2); too short or not accurately looking at the stimuli (*N* = 4); detection problems with the eye tracker due to droopy eyes (*N* = 1), third eyelids (*N* = 1), or uncertain pupil edges (*N* = 1). From a total of 66 dogs, 41 completed the training, of which five dogs had discontinued training due to decisions by the owner that had nothing to do with the training (completion ratio: 67.2%).

Out of these 41 dogs trained and tested with the big screen, 30 dogs learned to conduct eye-tracking tests on the small PC monitor, as well.

The dogs (*N = 41*) needed 15 training sessions, on average (range: 8 to 30), until they were able to conduct eye-tracking tests. The following training for eye-tracking tests with the small monitor (*N* = 30) lasted on average nine sessions and ranged from four to 12 sessions. Using different subsamples of these 41 dogs, between 2015 and 2017 we conducted 11 different eye-tracker tasks, with a minimum of 13 to a maximum of 38 participating dogs (Barber et al., 2017; Barber et al., 2016; Delay, 2016). A few studies that are not yet completed or published investigated the looking patterns of dogs when presented with, for example, videos of humans interacting with dogs or videos of humans hiding objects (Binderlehner, 2017).

**Fig. 5:**
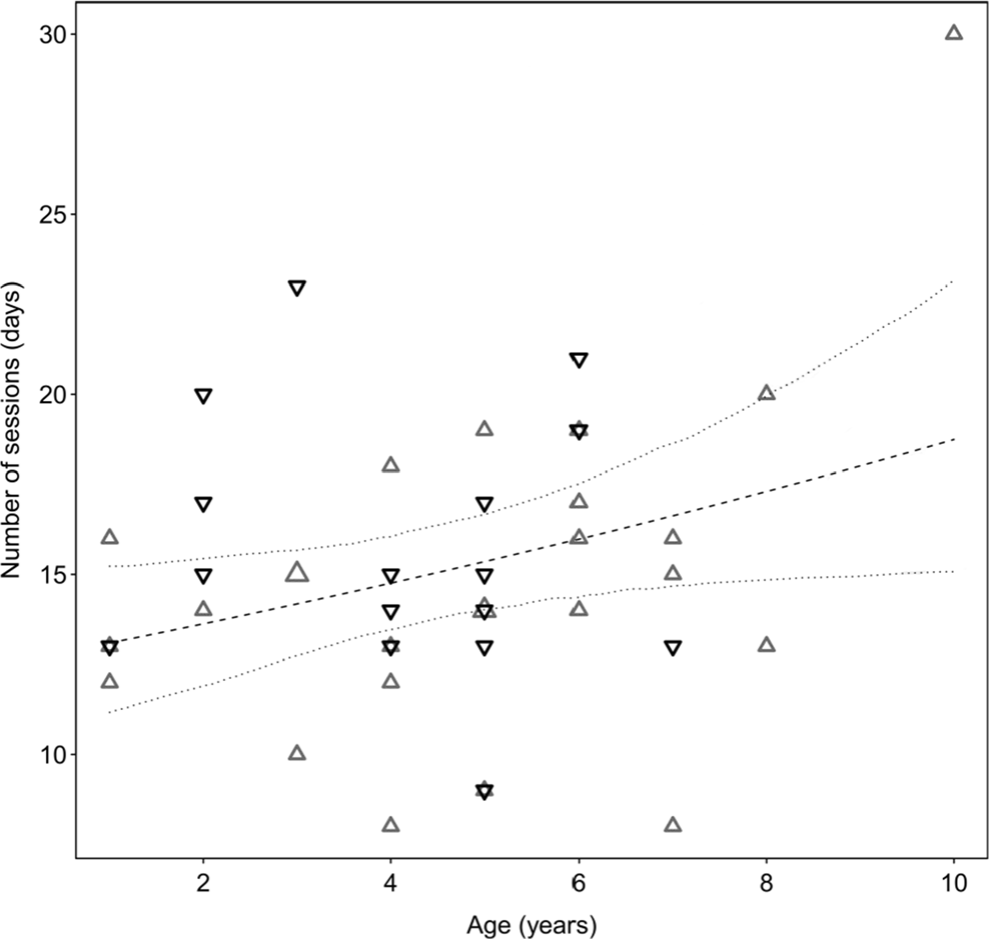
Relationship of age and duration of training (number of sessions until completion) of subjects trained for eye-tracking on the large back-projection screen (*N* = 41). Females are depicted with upright gray and males with upside-down black triangles. The sizes of the triangles represent the number of individuals per age and the number of sessions (*n* = 8 to 30). The dashed line shows the fitted values, and the upper and the lower finely dotted lines represent the confidence intervals

A main question concerning the use of dogs for eye-tracking tests was whether the age or the sex of the dogs had an effect on their training performance. However, we had only few very young (< 1 year, *N* = 6) and very old (10 years, *N* = 3) dogs from which to draw clear conclusions about their learning speed. The oldest dog in the eye-tracking training (Flora, 30 sessions) needed two times longer to complete the training than the average (15 sessions). Testing sex and age simultaneously in the full–null model comparison with the whole sample (*N* = 41) revealed that the full model was significantly different from the null model (*χ*^2^ = 6.381, *df* = 1, *p = .*011). Overall, when we tested the individual predictors, we found a significant age effect on the number of sessions needed for the big-screen training (*χ*^2^ = 0.073, *df* = 1, *p = .*041; see Table S5): Older dogs needed slightly more training sessions to reach the training goal than young dogs (Fig. 5). There was no significant effect of sex on the number of training sessions required (*χ*^2^ = 0.287, *df* = 1, *p = .*287).

When we ran the same analyses with the 30 dogs that completed eye-tracking training using the small computer monitor, there was no significant difference between the full and null models (*χ*^2^ = 0.170, *df* = 1, *p = .*679). We found only a weak trend for an effect of age on the number of training sessions needed (*χ*^2^ = 0.051, *df* = 1, *p = .*054), and again no effect of sex (*χ*^2^ = 0.497, *df* = 1, *p = .*495; see Table S6).

### fMRI training and testing

To date, 21 (including one deaf dog) out of 24 dogs were trained to be scanned while they stayed unrestrained and motionless with their head in the human knee coil in the MRI scanner bore. Although the deaf dog did not deviate from the behavior of all the other dogs in the scanner, it was not included in the analysis because of the omitted noise habituation training (12 training sessions). From a total of 24 dogs, 21 were trained successfully; the three remaining dogs are still in the mock-scanner pretraining. Therefore, we might finally achieve a 100% completion ratio.

Nineteen dogs were scanned with the 4-min structural scan sequence and produced accurate anatomical brain scans (see, e.g., Fig. S4). The number of attempts necessary to get the structural scan varied between 1 and 35 (mean: 6.5) within 1–13 sessions (mean: 3.3).

Seven dogs could already be tested successfully in three different fMRI tasks. During the tasks, the dogs lay motionless in the scanner bore and watched the presented visual stimuli attentively. They perceived dynamic pictures of their caregivers and of unfamiliar and familiar persons showing different facial expressions.

On average, the dogs (*N* = 20; eight males, 12 females) needed 21 sessions (range: 8 to 36) to complete the mock-scanner pretraining. The highest number of sessions was needed by the only dog that started at puppy age (3 months), whereas the oldest two dogs (Emily and Aeden, both 10 years old) performed at or below the average (21 and 19 sessions).

Although six dogs in our sample were very noise-sensitive—for instance, toward fireworks, shooting, or thunderstorms—we nevertheless could train them successfully for fMRI scanning following our training program.

Analyzing the complete data set, comprising all 20 dogs, the full–null model comparison that tested sex and age simultaneously revealed no significance (*p = .*179). Overall, we found a significant effect of neither age (*p = .*174) nor sex (*p = .*284; Table S7).

**Fig. 6:**
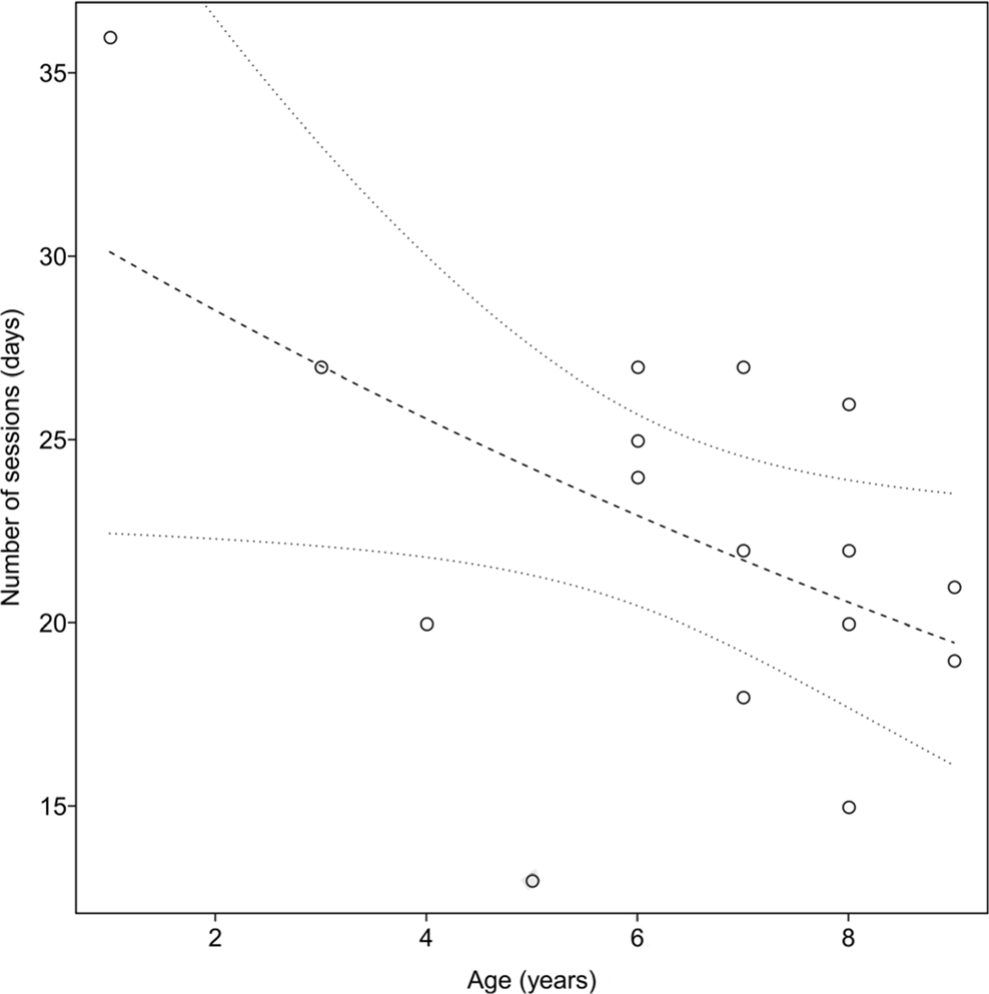
Relationship of age and duration of training (number of sessions until completion) of only the Border Collies trained for fMRI with the mock scanner (*N* = 16). The dashed line shows the fitted values, and the upper and lower finely dotted lines represent the confidence intervals

Testing a subset of the data, comprising only the Border Collies (*N* = 16), the full model was significantly different when compared to the null model (*p* < .001). Overall, we found a significant effect of age on the number of training sessions required (*p = .*026; see Fig. 6, Table S7). Sex was not significant in either data set (all dogs, *p = .*284; Border Collies, *p = .*978).

**Fig. 7:**
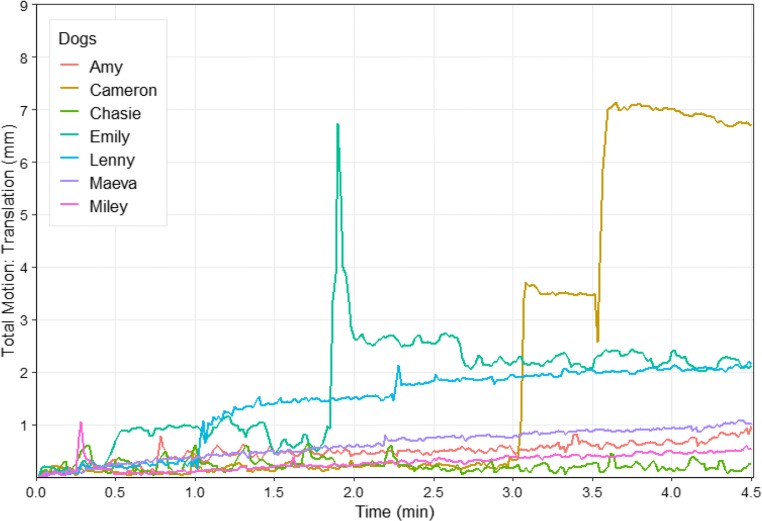
Total motion of seven dogs during their first attempt at actual data collection. The total translation motion (*x*, left–right; *y*, forward-backward; *z*, up-down) was calculated as the Euclidean distance (the square root of *x*^2^ + *y*^2^ + *z*^2^) from the start position. Movement parameters were generated using the Statistical Parametric Mapping software package (SPM12, Wellcome Trust Centre for Neuroimaging, UCL, London, UK) and plotted using the R package ggplot2 (Wickham, 2016). Total rotational motion is provided in Fig. S6

A main criterion for the success of the training program was the performance of the dogs during scanning. Would they indeed remain motionless during the whole scanning procedure? Figure 7 shows the movements of all dogs during their first attempt at data collection with a multiband echo-planar imaging sequence with 24 axial slices (MB factor 2, GRAPPA factor 2, interleaved acquisition, voxel size: 1.5 × 1.5 × 2 mm^3^, 0.4-mm slice gap. TR/TE = 1,000/38 ms, field of view = 144 × 144 × 58 mm^3^, flip angle = 61°). For most of the time (duration = 4.5 min), the movements of the dogs remained below 2 mm. Only two dogs showed strong movements: Cameron left the scanner after 3.5 min, and Emily adjusted her head position after approximately 2 min. An example of how a dog improved from the early training stage to the final training session before actual data collection is provided in Fig. S5.

## Discussion

We have described two programs for the training of (pet) dogs to be used as subjects in experiments that apply either eye-tracking or fMRI. The main goal of both programs is to end with subjects that are able to lie (or sit or stand) motionless for several minutes while being awake and fully attentive toward visual or other sensory stimuli. This goal has been achieved in a manner that is effective, efficient, and welfare-friendly—that is, highly successful in terms of both completion ratio (eye-tracking: 67.2%, fMRI: 100%) and performance during testing—but at the same time meets the highest ethical standards of dog training. The latter means training without any threats, force, positive punishment, or any other compromises of the dog’s well-being. By reporting data from 41 dogs that successfully participated in eye-tracking training, and 24 dogs in fMRI training, we have provided sufficient evidence for the quality and efficiency of our training methods. We therefore recommend our training programs to future scientists who start to apply eye-tracking or fMRI for their investigations of canine behavior and cognition.

The reliability of the training method is reflected in the findings that we could train males and females with similar success, despite the fact that some former studies have reported sex differences in the trainability of certain breeds (Serpell & Hsu, 2005). Interestingly, the age effects we found were also marginal and contradictory in the two tasks. Nevertheless, it is important to note that the dogs’ ages did not vary broadly in our relatively small samples; therefore it is feasible to assume that the training takes longer in young (1–3 years old), as well as in older or senior (8–10 years old), dogs that, for different reasons, might not be able to concentrate for a longer time. Pointing in this direction, Wallis et al. (2014), testing a larger cohort of 145 dogs, found young and senior-aged dogs to be more distractible than middle-aged (3–6 years) dogs. As with older humans, it could be that due to developmental (neuroplastic) changes, older dogs learn more slowly than younger and middle-aged dogs (Lillard & Erisir, 2011). Finally, although again in a low number, we also trained noise-sensitive dogs successfully to lie calm in the MRI scanner. With regard to the quality of our stillness training, the most telling piece of the results is that the movements of most dogs (four out of seven) remained below 1 mm throughout scanning periods of up to 4.5 min.

Our training program is composed of traditional operant-conditioning techniques, systematic desensitization, and habituation. Desensitization and habituation were used to gradually get the dogs used to the narrow, noisy, and moving environment and to wearing human ear plugs, so that ultimately they were relaxed and concentrated in these situations and showed no undesired behaviors. At the same time, using operant conditioning, the probability of desired behaviors—for instance, keeping the head on the chin rest or lying still while the table was moved—was increased by using rewards such as food or verbal praise. This type of reward-based training is very effective in training dogs to perform basic obedience behaviors (e.g., “sit”), and it is therefore the most commonly used training method in canine research. The main behavior in the tasks presented here (“lie still”) is very similar to one of the main obedience tasks for dogs (“lie”), and therefore it was not surprising that it emerged quickly on the basis of operant conditioning. The challenge, rather, was to extend this behavior over a period of minutes, which we managed by means of steps and carefully forming the correct behavior and increasing the duration of the rest time. It was crucial to train the dogs to leave the head in the exact same position in the coil right from the start. This behavior needed to be established by several repetitions over sessions.

As we mentioned in the introduction, former studies used other training methods and other human coils to prepare the dogs for fMRI studies. In Budapest, in order to prepare the dogs for the awake fMRI testing, researchers have used a mixture of conditioning and social learning, the latter being a modified version of the model–rival technique (Andics et al., 2014). This method had been derived from social modeling theories (Bandura, 1971; Mowrer, 1960), first to train vocal patterns (antiphonal duetting) in grey parrots (Todt, 1975). Pepperberg (1981, 1994, 1999) has championed this technique to teach Alex, an African grey parrot, how to recognize objects by some distinguishing features, such as their name, color, size, shape and quantity, by observing a trainer and a potential competitor engage in conversation about these features. In those experiments, one human is the exclusive cooperative partner (the trainer) of the parrot, while another human acts both as a model for the bird’s responses and as a rival for the trainer’s attention.

In Budapest, novice dogs were allowed in the beginning of their training to participate off-leash in the scanner room during the training session of a familiar dog (Andics et al., 2014). When the model (the dog in the scanner) was praised and rewarded by the trainer and both caregivers, the novice dog was ignored. Importantly, this method requires having two dogs from the same household or “friends” from the dog school (Andics et al., 2014; see the supplementary material). If dogs do not know each other, the model–rival method may cause aggressive attacks between the “rivals,” as has been found in lar gibbons (Heyes & Galef, 1996). Although dogs are derived from a highly social species, wolves, they are less tolerant of proximity during feeding on a monopolizable food source than were their wild ancestors (Range, Ritter, & Virányi, 2015). As such, using the model–rival method may impose some limitations on the selection of the subjects. Beyond this, however, there is no reason to assume a difference in the efficiency of this method and our more classical conditioning techniques. No direct comparison of these training programs is possible at this stage, for instance because in our study, about half of the dogs were trained together with a second dog from the same household. Although the second dog in the training session was not allowed to move freely in the room but was required to wait in close vicinity to the caregiver in the corner of the room until the training session of the first dog was finished, we cannot exclude that observing the training sessions of the fellow dog improved the performance of these dogs. In our training sample we had seven caregivers that participated with two dogs at the same time in the fMRI training. In three dog pairs one dog was already eye-tracker trained and it turned out that this dog needed fewer training sessions (six or seven) than the other one. This might show that the former eye-tracker training influenced the fMRI training performance. These dogs knew already how to perform calm tasks and the trainer. Among the pairs of dogs that had both been eye-tracker trained before (*N* = 4), only one pair needed almost the same amount of training sessions. In three cases, there was still a big difference in the training sessions required (five to seven). The training performance seemed to be based on the individual. In all cases, the younger dog needed more sessions than the older one. But since the age difference ranged from one to six years and the sample size was very small, we cannot draw any strong conclusions about what influenced the training performance. Nevertheless, other studies have shown that conditioning can be similarly or more effective than the model–rival technique (Cracknell, Mills, & Kaulfuss, 2008). McKinley and Young (2003) found no difference in the learning speed of pet dogs trained by either technique to solve a retrieval-selection task—that is, to correctly select the commanded object to bring to the experimenter from a group of similar objects.

In agreement with all groups that have so far trained dogs to remain motionless in either eye-tracking or MRI scanning, we propose that training is strictly done with positive reinforcement. As reward, we used both treats (food) and social praise, with the latter being healthier and at least as affective as food (Cook, Prichard, Spivak, & Berns, 2016). The dogs should always be highly motivated to participate, enter the training and testing environments voluntarily and enjoy training, which only demands actions from the dogs’ natural behavioral repertoire like sitting, lying, walking, and watching. If the dog does not like the procedures or is even anxious, the tests would no longer be valid. Therefore, we arranged our training and testing setting in a way that the dogs felt comfortable at any time and were motivated and able to learn. We adapted the training steps according to the dogs’ well-being and individual learning speed. If we noticed any signs of discomfort or distress in the dog’s behavior or body language we stopped immediately and changed the situation to continue positively with the dog. This requires the trainer and experimenter to be highly qualified for dog training and experienced for detecting stress signals in dogs. And finally, although dogs have different personalities, the training regime has to be standardized by using a strict and transparent training protocol.

The most important features of training to be effective are its accuracy and reliability. Many trainers therefore use a clicker to shape the dog’s behavior by eliciting prompt and correct response to commands (Feng, Howell, & Bennett, 2017, 2018; Lindsay, 2000; Tillman, 2000). The advantage of this classically conditioned secondary reinforcer (Skinner, 1938) is the exact timing (“split-second precision”) and the high reproducibility (Pryor, 1999, 2005). Rather than using verbal signals that may vary in loudness, length, pitch etc., the clicker produces always the exact same conspicuous acoustic signal (the metallic “click”). And, in contrast to the primary reinforcer—for example, the food reward—it can be delivered in the instant the correct action occurs, thereby marking the event in memory (Lieberman, McIntosh, & Thomas, 1979) and filling in the temporal delay between the behavioral response and the primary reinforcer (Kaplan & Hearst, 1982). However, the beneficial effect of this predictor signal would be rather small if it were not performed well (precise timing) or if alternative methods were performed with high accuracy by professional dog trainers. In fact, the results of studies comparing different training methods have shown no advantage related to clicker use, as dogs trained with the clicker learned the new behavior as fast as dogs trained with a word as the secondary reinforcer, or with food alone (Chiandetti, Avella, Fongaro, & Cerri, 2016; Feng, Howell, & Bennett, 2016; Smith & Davis, 2008). Therefore, whether or not future researchers prefer to use a clicker is less important than having a straightforward training plan with clear performance criteria and without losing sight of the main objective, which is to keep the dog motivated and eager to learn. Above all, the use of reward-based training appears to be the most beneficial system in terms of both the training objectives and the dogs’ welfare, since it is linked to enhanced learning and a balanced, healthy dog–scientist relationship (Rooney & Cowan, 2011). In contrast, the use of aversive-based methods is correlated with indicators of compromised welfare in dogs—that is, stress-related behaviors during training and problematic behaviors such as fear and aggression (Beerda, Schilder, van Hooff, & de Vries, 1997; Fernandes, Olsson, & de Castro, 2017; Hiby, Rooney, & Bradshaw, 2004).

In conclusion, we suggest that the investigation of certain cognitive abilities of dogs under rigorous experimental conditions and by using the latest and most sophisticated technical procedures (eye-tracking and fMRI) requires equally sophisticated preparation, in terms of dog training and habituation. Here we propose a training regime that is perfectly suited to train dogs in the required skills—motionless watching and mentally processing of visual stimuli—with a high probability of success and without threats, force, or punishment. The use of carefully progressing, reward-based training appears to be the most beneficial approach for both the dog’s welfare and the scientific outcome.

## Electronic supplementary material

ESM 1 (DOCX 670 kb)
