# Supplementary material for: Training pet dogs for eye-tracking and awake fMRI
Source: Behav Res Methods. 2019 Jul 16;52(2):838–56. doi: 10.3758/s13428-019-01281-7 (PMC7148272; doi:10.3758/s13428-019-01281-7)
Supplement: Supplementary file 1 — (DOCX 670 kb) [file 13428_2019_1281_MOESM1_ESM.docx]

**SUPPLEMENTARY MATERIAL**

***Table S1***

*Overview of all successfully trained dogs for the use of the eye-tracker*

| **no.** | **dog** | **breed** | **sex** | **age in 2015 (in years)** | **trained in (year)** | **trained for big screen** | **trained for small screen** |
| --- | --- | --- | --- | --- | --- | --- | --- |
| **1** | **Aeden** | Border Collie | m | 7 | 2014-2015 | x | x |
| **2** | **Akin** | Rhodesian Ridgeback | m | 6 | 2014-2015 | x | x |
| **3** | **Akina** | Akita | f | 7 | 2014-2015 | x | x |
| **4** | **Apryl** | Border Collie | f | 6 | 2014-2015 | x | x |
| **5** | **Arielle** | Border Collie | f | 1 | 2014-2015 | x | x |
| **6** | **Aslan** | Staffordshire Terrier | m | 4 | 2014-2015 | x |  |
| **7** | **Baily** | Border Collie | f | 5 | 2014-2015 | x | x |
| **8** | **Balian** | Border Collie | m | 5 | 2014-2015 | x | x |
| **9** | **Bandit** | Border Collie | m | 4 | 2014-2015 | x |  |
| **10** | **Beauty** | Border Collie | f | 5 | 2014-2015 | x |  |
| **11** | **Cameron** | Border Collie | m | 3 | 2014-2015 | x | x |
| **12** | **Carlisle** | Border Collie | m | 4 | 2014-2015 | x | x |
| **13** | **Casper** | Border Collie | m | 2 | 2014-2015 | x |  |
| **14** | **Chasie** | Border Collie | f | 6 | 2014-2015 | x | x |
| **15** | **Cheynna** | Australian shepherd | f | 1 | 2014-2015 | x |  |
| **16** | **Dexter** | Border Collie | m | 5 | 2014-2015 | x |  |
| **17** | **Emily** | Border Collie | f | 7 | 2014-2015 | x | x |
| **18** | **Fenja** | Border Collie | f | 8 | 2014-2015 | x |  |
| **19** | **Flora** | Golden Retriever | f | 10 | 2014-2015 | x |  |
| **20** | **Gatsby** | Border Collie | m | 5 | 2014-2015 | x | x |
| **21** | **Hagrid** | mixbreed | m | 5 | 2014-2015 | x | x |
| **22** | **Iken** | Border Collie | m | 2 | 2014-2015 | x |  |
| **23** | **Kayleigh** | Border Collie | f | 4 | 2014-2015 | x | x |
| **24** | **Keksi** | mixbreed | f | 3 | 2014-2015 | x | x |
| **25** | **Lara** | Border Collie | f | 3 | 2014-2015 | x | x |
| **26** | **Lenny** | Border Collie | m | 6 | 2014-2015 | x |  |
| **27** | **Linus** | Australian Shepherd | m | 1 | 2014-2015 | x | x |
| **28** | **Luna** | Sibirian Husky | f | 3 | 2014-2015 | x | x |
| **29** | **Miley** | Border Collie | f | 6 | 2014-2015 | x |  |
| **30** | **Monty** | Border Collie | m | 5 | 2014-2015 | x |  |
| **31** | **Muffin** | mixbreed | f | 4 | 2014-2015 | x |  |
| **32** | **Mulan** | mixbreed | f | 5 | 2014-2015 | x | x |
| **33** | **Müsli** | Border Collie | m | 2 | 2014-2015 | x | x |
| **34** | **Roxie** | mixbreed | f | 7 | 2014-2015 | x | x |
| **35** | **Schoko** | Magya Viszla | f | 5 | 2014-2015 | x | x |
| **36** | **Tiara** | Border Collie | f | 2 | 2014-2015 | x |  |
| **37** | **Tika** | mixbreed | f | 8 | 2014-2015 | x | x |
| **38** | **Tini** | Boxer | f | 4 | 2014-2015 | x | x |
| **39** | **Tuukka** | mixbreed | f | 1 | 2014-2015 | x | x |
| **40** | **Ziva** | Border Collie | f | 4 | 2014-2015 | x | x |
| **41** | **Zuri** | Rhodesian Ridgeback | f | 6 | 2014-2015 | x | x |

***Table S2:***

*Overview of all successfully trained dogs for the use of the eye-tracker with the small monitor and their needed training sessions*

| **no.** | **dog** | **breed** | **age in 2016** | **sessions** |
| --- | --- | --- | --- | --- |
| **1** | **Akina** | Akita Inu | 7 | 10 |
| **2** | **Mulan** | mixbreed | 4 | 8 |
| **3** | **Hagrid** | mixbreed | 4 | 8 |
| **4** | **Roxie** | mixbreed | 8 | 8 |
| **5** | **Schoko** | Magyar Vizsla | 4 | 6 |
| **6** | **Lara** | Border Collie | 6 | 12 |
| **7** | **Tini** | Boxer | 3 | 6 |
| **8** | **Akin** | Rhodesian Ridgeback | 7 | 11 |
| **9** | **Zuri** | Rhodesian Ridgeback | 5 | 11 |
| **10** | **Müsli** | Border Collie | 3 | 9 |
| **11** | **Chasie** | Border Collie | 7 | 12 |
| **12** | **Gatsby** | Border Collie | 6 | 10 |
| **13** | **Kayleigh** | Border Collie | 5 | 12 |
| **14** | **Apryl** | Border Collie | 7 | 9 |
| **15** | **Aeden** | Border Collie | 8 | 13 |
| **16** | **Ziva** | Border Collie | 5 | 7 |
| **17** | **Emily** | Border Collie | 8 | 6 |
| **18** | **Baily** | Border Collie | 5 | 11 |
| **19** | **Amy8** | Border Collie | 6 | 9 |
| **20** | **Maeva** | mixbreed | 5 | 13 |
| **21** | **Tika** | Sibirian Husky | 7 | 10 |
| **22** | **Linus** | Australian Shepherd | 1 | 9 |
| **23** | **Cameron** | Border Collie | 4 | 9 |
| **24** | **Keksi** | mixbreed | 2 | 9 |
| **25** | **Leopold** | Brabanter Griffon | 7 | 9 |
| **26** | **Carlisle** | Border Collie | 5 | 7 |
| **27** | **Balian** | Border Collie | 6 | 11 |
| **28** | **Luna** | Sibirian Husky | 5 | 8 |
| **29** | **Tuukka** | mixbreed | 2 | 5 |
| **30** | **Arielle** | Border Collie | 1 | 4 |

***Table S3***

*Overview of all successfully trained dogs for the use of the MRI scanner*

| **no.** | **dog** | | **breed** | **sex** | **age in 2016/ 2017 (in years)** | **trained in (year)** | **in MRI scanner training** | **in mock scanner training** |
| --- | --- | --- | --- | --- | --- | --- | --- | --- |
| **1** | **Aeden** | Border Collie | | m | 9 | 2017 | x |  |
| **2** | **Amy8** | Border Collie | | f | 7 | 2017 | x |  |
| **3** | **Apryl** | Border Collie | | f | 8 | 2017 | x |  |
| **4** | **Balian** | Border Collie | | m | 7 | 2017 | x |  |
| **5** | **Cameron** | Border Collie | | m | 5 | 2017 | x |  |
| **6** | **Carlisle** | Border Collie | | m | 6 | 2017 | x |  |
| **7** | **Chasie** | Border Collie | | f | 8 | 2017 | x |  |
| **8** | **Cliff** | Border Collie | | m | 3 | 2017 | x |  |
| **9** | **Emily** | Border Collie | | f | 9 | 2017 | x |  |
| **10** | **Gatsby** | Border Collie | | m | 7 | 2017 | x |  |
| **11** | **Jace** | Border Collie | | m | 1 | 2017 | x |  |
| **12** | **Kayleigh** | Border Collie | | f | 6 | 2017 | x |  |
| **13** | **Kiki** | mixbreed | | f | 2 | 2017 | x |  |
| **14** | **Lenny** | Border Collie | | m | 8 | 2017 | x |  |
| **15** | **Linus** | Australian Shepherd | | m | 3 | 2017 | x |  |
| **16** | **Luise**  **(deaf)** | mixbreed | | f | 4 | 2017 | x |  |
| **17** | **Maeva** | mixbreed | | f | 6 | 2017 | x |  |
| **18** | **Miley** | Border Collie | | f | 8 | 2017 | x |  |
| **19** | **Schoko** | Magya Viszla | | f | 7 | 2017 |  | x |
| **20** | **Sunny** | Border Collie | | f | 1 | 2017 |  | x |
| **21** | **Tiara** | Border Collie | | f | 4 | 2017 | x |  |
| **22** | **Tini** | Boxer | | f | 6 | 2017 | x |  |
| **23** | **Ziva** | Border Collie | | f | 6 | 2017 | x |  |
| **24** | **Zuri** | Rhodesian Ridgeback | | f | 8 | 2017 |  | x |

***Table S4***

*Age and needed mock scanner sessions of MRI trained dogs*

| **no.** | **dog** | **age** | **sessions** |
| --- | --- | --- | --- |
| **1** | **Maeva** | 7 | 8 |
| **2** | **Luise (deaf)** | 4 | 12 |
| **3** | **Cameron** | 6 | 13 |
| **4** | **Miley** | 9 | 15 |
| **5** | **Balian** | 8 | 18 |
| **6** | **Emily** | 10 | 19 |
| **7** | **Kiki** | 3 | 19 |
| **8** | **Tiara** | 5 | 20 |
| **9** | **Apryl** | 9 | 20 |
| **10** | **Aeden** | 10 | 21 |
| **11** | **Lenny** | 9 | 22 |
| **12** | **Amy8** | 8 | 22 |
| **13** | **Tini** | 7 | 22 |
| **14** | **Carlisle** | 7 | 24 |
| **15** | **Kayleigh** | 7 | 25 |
| **16** | **Linus** | 4 | 25 |
| **17** | **Chasie** | 9 | 26 |
| **18** | **Ziva** | 7 | 27 |
| **19** | **Gatsby** | 8 | 27 |
| **20** | **Cliff** | 4 | 27 |
| **21** | **Jace** | 1 | 36 |

***Table S5***

*ET big screen data set: Results of the GLMM investigating the effect of age and sex on the number of training sessions for all dogs (N=41)*

| **term** | **estimate** | **SE** | **Lower CL** | **Upper CL** | **χ²** | **df** | **P-value** | **Min^c^** | **Max^c^** |
| --- | --- | --- | --- | --- | --- | --- | --- | --- | --- |
| Intercept | 2.497 | 0.111 | 2.264 | 2.689 |  |  | NI^a^ | 2.457 | 2.601 |
| Age | 0.040 | 0.020 | 0.002 | 0.080 | 0.073 | 1 | 0.041 | 0.011 | 0.062 |
| Sexm^b^ | 0.089 | 0.083 | -0.080 | 0.246 | 0.287 | 1 | 0.287 | -0.002 | 0.107 |

^a^ not indicated because of its limited interpretation

^b^ sex was manually dummy coded and centered with the females as reference

^c^ Minimum and maximum of model estimates when dropping each case one at a time

***Table S6***

*ET small screen data set: Results of the GLMM investigating the effect of age and sex on the number of training sessions for the small screen trained dogs (N=30)*

| **term** | **estimate** | **SE** | **Lower CL** | **Upper CL** | **χ²** | **df** | **P-value** |
| --- | --- | --- | --- | --- | --- | --- | --- |
| Intercept | 1.857 | 0.185 | 1.485 | 2.210 |  |  | NI^a^ |
| Age | 0.061 | 0.032 | -0.005 | 0.125 | 0.051 | 1 | 0.054 |
| Sexm^b^ | 0.086 | 0.127 | -0.180 | 0.320 | 0.497 | 1 | 0.495 |

^a^ not indicated because of its limited interpretation

^b^ sex was manually dummy coded and centered with the females as reference

***Table S7***

*fMRI data set: Results of the GLM investigating the effect of age and sex on the number of training sessions for all dogs (N=20) and a subset of only Border Collies (BC, N=16)*

| **term** | **estimate** | **SE** | **Lower CL** | **Upper CL** | **z-value** | **P-value** | **Min^c^** | **Max^c^** |
| --- | --- | --- | --- | --- | --- | --- | --- | --- |
| **All dogs** | | | | | | |  |  |
| Intercept | 3.220 | 0.171 | 2.881 | 3.552 | 18.834 | NI^a^ | 3.114 | 3.363 |
| Age | -0.033 | 0.024 | -0.081 | 0.015 | -1.359 | 0.174 | -0.056 | -0.021 |
| Sexm^b^ | 0.120 | 0.112 | -0.101 | 0.341 | 1.071 | 0.284 | 0.058 | 0.195 |
| **Only BC** | | | | | | |  |  |
| Intercept | 3.458 | 0.185 | 3.091 | 3.815- | 18.730 | NI^a^ | 3.318 | 3.667 |
| Age | -0.055 | 0.024 | -0.102 | -0.006 | -2.228 | 0.026 | -0.085 | -0.040 |
| Sexm^b^ | 0.003 | 0.111 | -0.215 | 0.221 | 0.028 | 0.978 | -0.067 | 0.068 |

^a^ not indicated because of its limited interpretation

^b^ sex was manually dummy coded and centered with the females as reference

^c^ Minimum and maximum of model estimates when dropping each case one at a time

**
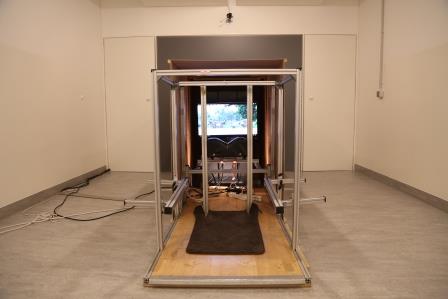
**

**
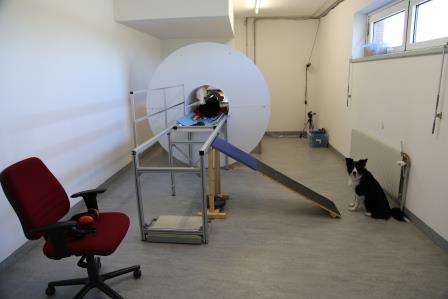
*Figure: S1:*** View of the eye-tracking room in the Clever Dog Lab of the University of Veterinary Medicine Vienna

***Figure S2:*** View of the mock scanner room in the Clever Dog Lab of the University of Veterinary Medicine Vienna

**
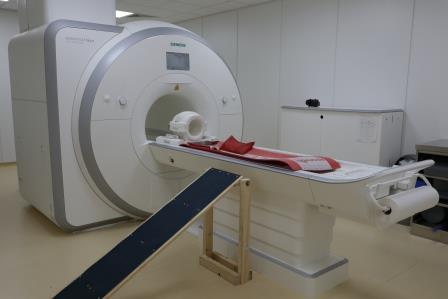
**

***Figure S3*:** View of the MRI scanner room in the MR Center of the University of Vienna

| 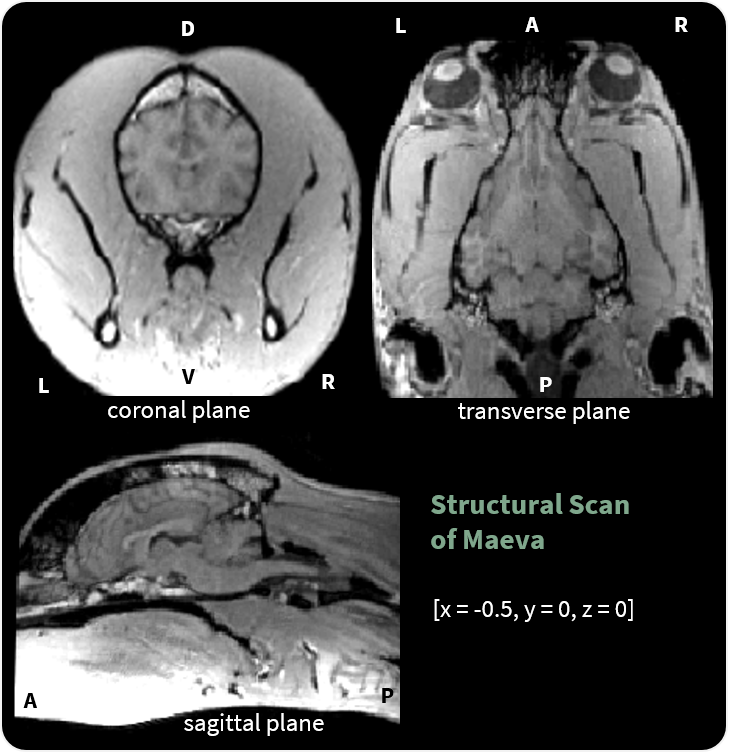 ***Figure S4:*** Structural image of Maeva, an eight-year-old female mixbreed. The structural image was manually reoriented using the SPM *Display* function and the anterior commissure (AC) was set as origin. Coordinates refer to MNI stereotactic space (P posterior, A anterior, D dorsal, V ventral, L left, and R right). Data acquisition time is 3:21 min (voxel size: 0.7 x 0.7 x 0.7 mm, TR/TE = 38/100 ms, FoV = 230 mm^3^) and the scan has been successfully acquired after 4 training sessions at the MR scanner. |
| --- |

| 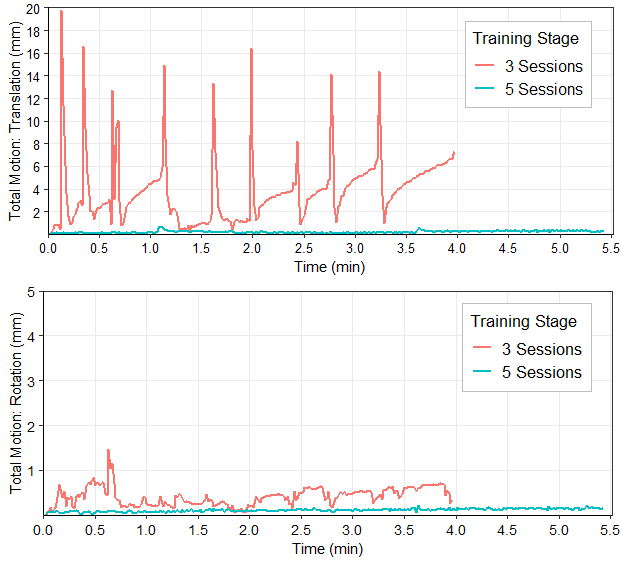 |  |
| --- | --- |
| ***Figure S5:*** The graphs depict the decreased movement of Maeva over her training course by comparing the first functional scan at an early training stage (3 scanner sessions) to the fifth and final training session (simulated test task, 5.5min) before actual data collection. Maeva’s movements decreased over the training course to a minimum of 1 mm while the duration of the scans increased. Total translation motion (x: left-right, y: forward-backward, z: up-down) depicted in the first graph (above) was calculated as in Figure 7. The graph below refers to the total rotation (pitch, roll, yaw) transformed from radiant (rad) to millimetre (mm) by multiplying rad with the estimated radius of the brain (Power et al., 2014) derived from measurements using ITK-snap (Yushkevich et al., 2006); and finally calculated as the Euclidean distance from the start point. Movement parameters were generated using SPM12 and plotted using ggplot2 (Wickham, 2016). For display purposes, the y-axis scales of the graphs differ. |  |
| ***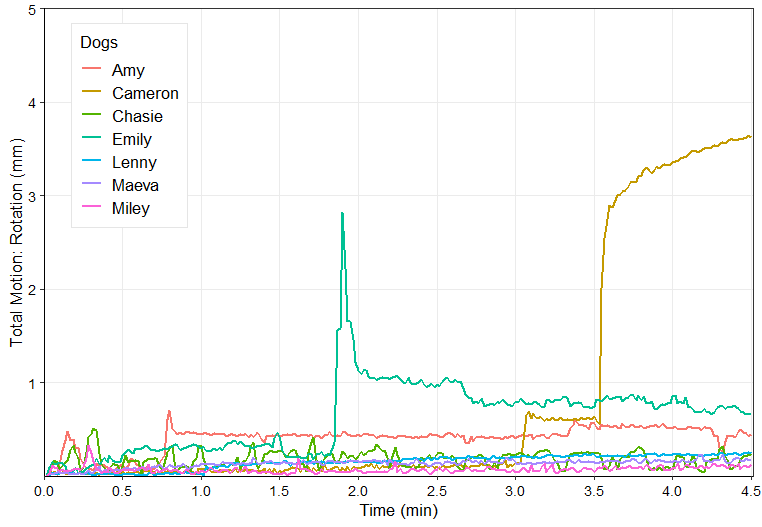*** | |
| ***Figure S6:*** The movement parameters depict the dogs’ rotational movements (pitch, roll, yaw) at their first data collection attempt (4.5 min run). Almost all dogs moved below 1 mm, Cameron exited the scanner bore towards the end of data collection. | |


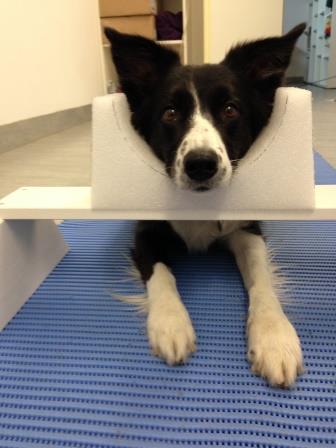

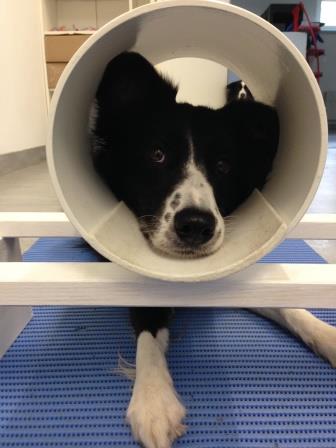

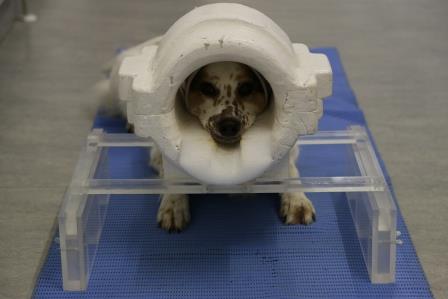


***Figure S7:*** Possible practice chin rests for caregivers (left: open, middle: closed) and the mock human knee coil (right).

**References**

Power, J. D., Mitra, A., Laumann, T. O., Snyder, A. Z., Schlaggar, B. L., & Petersen, S. E. (2014). Methods to detect, characterize, and remove motion artifact in resting state fMRI. *NeuroImage*, *84*, 320–341. doi: 10.1016/j.neuroimage.2013.08.048

Wickham, H. (2016). ggplot2: elegant graphics for data analysis. Springer.

Yushkevich, P. A., Piven, J., Hazlett, H. C., Smith, R. G., Ho, S., Gee, J. C., & Gerig, G. (2006). User-guided 3D active contour segmentation of anatomical structures: Significantly improved efficiency and reliability. *NeuroImage, 31*(3), 1116–1128.
